# Using Solution History to Control Hydrogel Properties of a Perylene Bisimide

**DOI:** 10.1002/chem.202301042

**Published:** 2023-05-10

**Authors:** Rebecca E. Ginesi, Nicholas R. Murray, Robert M. Dalgliesh, James Doutch, Emily R. Draper

**Affiliations:** ^1^ School of Chemistry University of Glasgow Glasgow, UK G12 8QQ UK; ^2^ ISIS Rutherford Appleton Laboratory Chilton Oxfordshire OX11 0QX UK

**Keywords:** gels, kinetics, low molecular weight gelators, perylene bisimide, self-assembly

## Abstract

pH dependence on water soluble aggregates is well‐known in the field of low molecular weight gelators (LMWGs), with different aggregates sometimes having very different properties depending on their final pH. This aggregation determines their applications and performance. Here, we investigate the pH dependence of perylene bisimide gels; initially solutions are formed at a high pH and gels form as the pH is decreased. We find it is not only the final pH but also the starting pH that can impact the resulting gel. We use small angle neutron scattering (SANS), rheology, ^1^H NMR spectroscopy and absorption spectroscopy to examine the effect of starting pH on gelation kinetics and final gel properties. Adjusting the solution from pH 9 (where there are few or no aggregates) to pH 6 results in the formation of different worm‐like micelles than the ones directly formed at pH 6, leading to again gels with different mechanical properties. This work highlights the importance of controlling the pH of solutions before gelation, but also opens up more possible morphologies and therefore more properties from the same molecule.

## Graphical abstract

**Gelation**: We demonstrate how one perylene bisimides gelator and one gelation trigger can form different hydrogels with different mechanical and optical properties. This is done by altering the starting pH and the history of the solution before it is gelled. This demonstrates the tunability of a single molecule using the same gelation conditions is possible, which is valuable in material design.

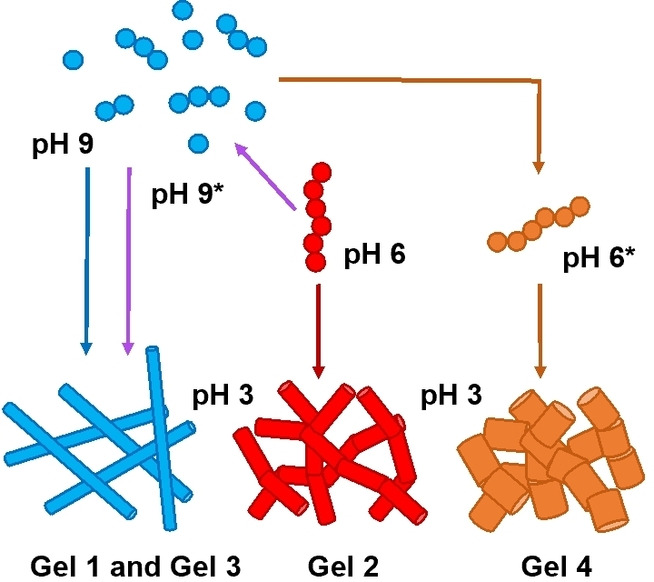

## Introduction

Low molecular weight gelators (LMWGs) can self‐assemble to form entangled gelatinous networks. These networks can be used for cell growth, drug delivery and storage, or as a reaction space, and filters, etc.^[1]^ This variety in applications is due to the pores that are formed and the flow of liquid through the solid‐like material. Often in these types of materials, the gel is trapping a material in the network whilst allowing others to pass through or providing a flexible mechanical substrate. Some molecules form gels that can also have an active role in the function of the material, such as in electronics,^[2]^ chromics,^[3]^ water‐splitting,^[4]^ photooxidation^[5]^ and sensing.^[6]^ Again, these make use of the liquid passing through the network whilst the active components, which in this case is the gel network itself, are immobilized and in higher concentration. In the cases of the active gel network, it has been reported that the fibre morphology and the network type affect the properties and performance of the materials for their intended outcome.^[7]^ External conditions have been seen to influence the structures that make up the gel network. These include factors such as temperature, solvents, additives, and pH. These influence morphology either by affecting the molecules themselves or changing the kinetics of assembly, which in turn changes the final aggregated form.[^7a^, ^8^] Often these assembly conditions may not be suitable for the final applications, such as high temperatures and the addition of salts or solvents can be detrimental for cells,^[1b]^ for example, or makes processing the materials very difficult, but it a very useful way of making different materials from the same molecule, rather than designing new molecules from scratch.

**Figure 1:**
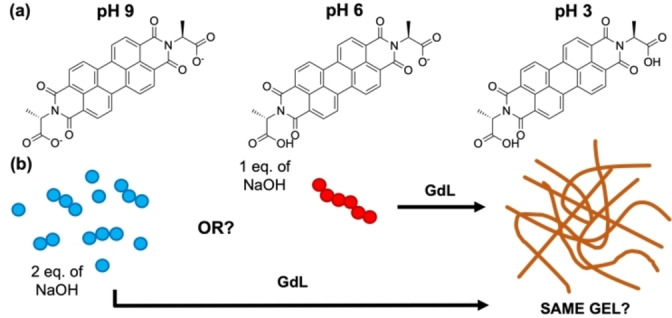
(a) Chemical structure of **PBI‐A** at the different pHs. (b) Cartoon highlighting the different method used to form gels in this study.

Perylene bisimides (PBIs) are an example of an active gel network and are commonly used in optoelectronic devices and light harvesting.[^1a^, ^3^, ^9^] PBIs can be easily derivatised with ionisable groups (e. g. amino acids) at the imide position.[^9b^, ^10^] The assembly of the PBIs is also affected by concentration of the PBIs in solution and the polarity of solvent, so different supramolecular structures could be formed with different properties.[^2^, ^11^] Such PBIs are soluble in water at high pH due to deprotonation of the terminal carboxylic acid groups.^[10c]^ When the pH decreases below the p*K*_a_ value of the gelator, these carboxylic groups become protonated and a hydrogel is formed.^[12]^ These PBIs have two apparent p*K*_a_ values associated with the protonation of the different carboxylic acid groups on each imide position, which in turn give different aggregated states due to solubility (Figure 1).^[13]^ We have previously shown that by using different amino acids in the imide position of the PBIs we could and form gels using glucono‐∂‐lactone (GdL). We have also seen that different aggregated structures form at different pHs. These differently aggregated states have different properties such as photoconductivity.[^1e^, ^13^, ^14^]

With one PBI appended with L–DOPA we were able to switch between a H‐type aggregate and a J‐type by adjusting the pH. The H‐type and the J‐type had different mechanical properties, with the J‐type not being able to form a gel upon lowering the pH.^[15]^ Crucially, the two states could be switched between them many times with no effect on the final properties.

Here we focus on **PBI‐A** which has interesting photoelectric behaviours due the formation of the radical anion with UV light.[^4a^, ^13^, ^16^] We examine how by changing the pre‐gelled solution pH (and therefore, the pre‐aggregated starting form of the material) and using the same pH trigger, we can control the kinetics of gelation and so the mechanical properties of the final gel. We examine the self‐assembly and gelation of two solutions of **PBI‐A** (Figure 1) one at pH 9, where the molecules are more soluble, and one at pH 6, which is in between the two “apparent” p*K*_a_ values of this gelator.

## Results and Discussion

**PBI‐A** was synthesised as previously reported.^[13]^ Stock solutions of the gelator were prepared at a concentration of 5 mg mL^−1^, adding either 1 or 2 equivalents of NaOH (0.1 M, aqueous) to give solutions with a starting pH of 6 and 9, respectively. Hydrogels were formed by adding an amount of GdL to give a gel with a final pH of 3.2 (Table S1, Supporting Information).

**Figure 2:**
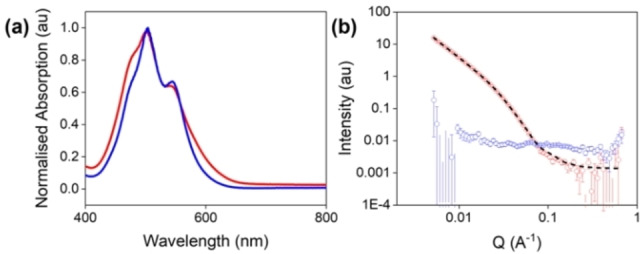
(a) UV‐vis absorption spectra of PBI−A solutions at pH 6 (red) and pH 9 (blue). (b) SANS from PBI−A solutions at pD 6 (red) and pD 9 (blue). Open circles show the data and black dashed lines show the fit.

First, we determined whether there were any differences in aggregation in the solutions at the different pH. Using absorbance spectroscopy, the peak ratios in the two solutions (at 490 and 540 nm) suggest a difference in molecular packing for each pH (Figure 2a).[^10c^, ^17^] The ratio of the peaks is 0.90:0.64 and 0.79:0.67 (490 : 540 nm) for pH 6 and 9, respectively. For solutions at pH 6, the spectrum was broader and the peaks were less defined, indicating that these solutions were more aggregated and viscosity suggested the presence of worm‐like micelles (Figure S1, Supporting Information).^[18]^ This behaviour was not observed at pH 9, suggesting that the PBI molecules were more dispersed or not forming persistent aggregates in solution. Differences were also seen in the fluorescence data with pH 6 and pH 9 having different ratios in the emission spectra and pH 6 being slightly more fluorescent than pH 9 (Figure S2, Supporting Information). Small angle neutron scattering (SANS) data confirms this, with little scattering seen.^[19]^ In comparison, scattering at pD 6 showed aggregates had formed with the SANS data best fitting to a flexible elliptical cylinder with a power law comparable with that previously reported with a radius of 43 Å (Figure 2b and Table S3, Supporting Information) again agreeing with the previous data.^[14]^

**Figure 3:**
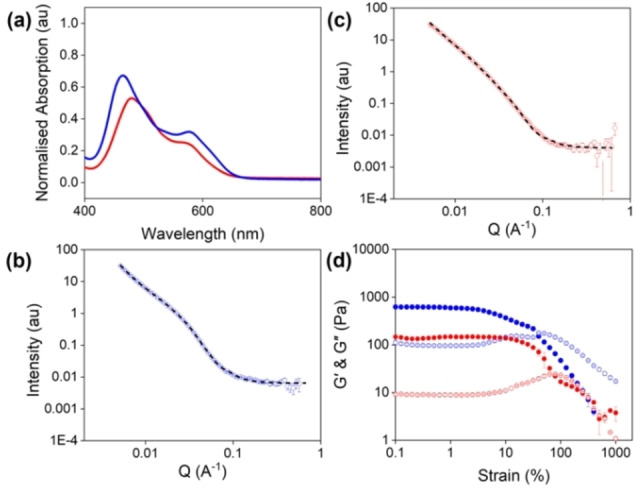
(a) UV‐vis absorption spectra of PBI−A gels formed from solutions starting at pH 6 (red) and pH 9 (blue). (b) and (c) SANS of gels formed from PBI−A solutions starting at pD 9 (blue) and pD 6 (red). Open circles show the data and black dashed lines show the fit. (d) Strain sweeps of PBI−A gels formed from solutions starting at pH 6, Gel 2 (red) and pH 9, Gel 1 (blue). Closed circles represent G′ and open circles represent G“. Data shown are averaged data for triplicate runs, with error bars being calculated using standard deviation.

Gelation was induced by adding GdL to these solutions. UV‐vis absorption spectroscopy showed significant differences in the molecular packing within the gels formed from the two starting pHs (Figure 3a). In both cases, the gels undergo a hypsochromic shift during gelation, suggesting the formation of H‐type aggregates and is most significant in gels formed from solutions starting at pH 9 (referred to as Gel 1) than from starting at pH 6 (referred to as Gel 2).^[10c]^

The scattering data from the Gel 1 fit to an elliptical cylinder with a power law with a radius of 61 Å (Figure 3b and Table S4, Supporting Information). In comparison, the data for the Gel 2 best fit to a flexible elliptical cylinder with a power law with a radius of 35 Å (Figure 3c and Table S5, Supporting Information). This clearly demonstrates the difference in the two gels formed. Rheological data also confirmed the differences with gel strength and stiffness being influenced by the starting pH of the solution (Figure 3d). Gel 1 were stiffer than the corresponding Gel 2 (G′ values at 0.01 % strain of 600 Pa and 170 Pa for Gel 1 and Gel 2, respectively). This increase in stiffness could be due to the formation of elliptical cylindrical fibres, which are more rigid than the flexible elliptical cylindrical fibres found in Gel 2. Furthermore, the Gel 2 were stronger, with a higher yield point (12.6 % strain compared to 3.2 % for the Gel 1). The increased flexibility in these gels could allow the gel to withstand higher stress as the fibres have more freedom of movement, allowing them to better respond to the force applied without breaking.^[20]^

**Figure 4:**
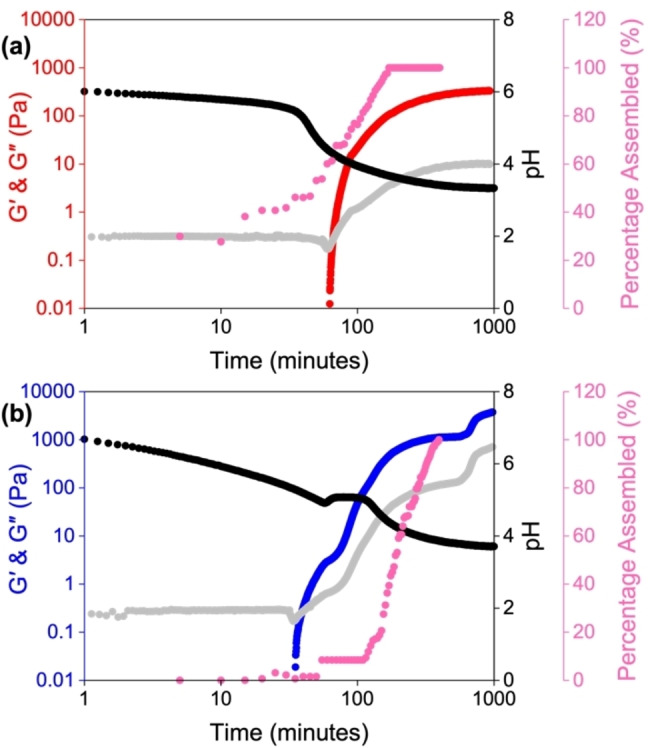
Plots showing the evolution of the gel networks starting at a pH of (a) 6 and (b) 9. The graphs show the development of G′ (red for a starting pH of 6 and blue for a starting pH of 9) and G“ (grey) with time and change in pH (black), and the change in percentage assembly (pink).

We hypothesised that the differences in the rheological and structural properties of the gels were due to different gelation kinetics since the final pH of the gels was the same. In theory, Gel 1 would have a more open pathway in the structures they could form upon gelation, whereas the pH 6 solution would have structures ‘locked in’ limiting the possibility of different aggregates upon its gelation. The controlled reproducible hydrolysis of GdL allows the gelation to be followed using numerous techniques, provided that the temperature and concentration are accurately controlled.^[21]^ Rheology, pH and ^1^H NMR data all showed clear differences in the self‐assembly process depending on the starting pH (Figure 4).^[21]^ Initially, we thought that this process would be the same irrespective of pH but would happen quicker when starting at pH 6, as the **PBI‐A** has already started to assemble. There are examples showing that changing the rate of hydrolysis and therefore the speed of gelation can influence final properties.^[22]^ However, instead we found that when starting at pH 9, there are three stages of self‐assembly, represented by the three plateaus in G′ and G“ (Figure 4b). In comparison, when starting at pH 6, the gelation is a single continuous process (Figure 4a). Using ^1^H NMR spectroscopy to follow this self‐assembly process we can calculate the self‐assembly by following the decrease in the integral values of the gelator.^[23]^

Upon addition of GdL to a solution of **PBI‐A** starting at pH 9, there is not much percentage assembly seen in the ^1^H NMR or the rheology, until 35 min, when G′ starts to increase, and the pH is 5.34, below its first “apparent” p*K*_a_ value (Figure 4a). After 75 min, there is an increase in pH before the pH and percentage assembled plateau. The pH begins to decrease again after 118 min, causing an increase in the percentage of molecules assembled. The percentage assembly reaches 100 % before the final plateau in G′ and G“. Such results indicate that the structures in solution may be elongating or thickening to entangle, which we have previously observed with other amino acid functionalised PBIs.^[24]^ The ^1^H NMR data also further suggests that the gelation process is a single process when starting at pH 6, as the percent assembly continuously increases over time. The percentage assembly is also 20 % at the beginning of the experiment, confirming the presence of pre‐assembled structures before gelation has been triggered. These data suggest a more complex explanation than simply a change in the rate of gelation and it is due to the different structures present in solution determining the gelation pathway.^[8g]^

**Figure 5:**
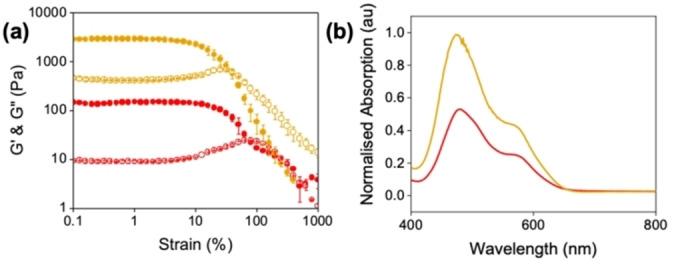
(a) Strain sweeps and (b) absorption data for PBI−A gels formed from solutions starting at pH 6 (Gel 2) (red) and pH 6* (Gel 4) (orange). Closed circles represent G′ and open circles represent G“. Data shown are averaged data for triplicate runs, with error bars being calculated using standard deviation.

We next investigated whether it was possible to change between the two differently aggregated states by adjusting the pH from either from pH 6 to 9 (referred to as pH 9*) or from pH 9 to 6 (referred to as pH 6*). To change from the singly deprotonated species found at pH 6 to the doubly deprotonated species found at pH 9, 1 equivalent of NaOH (0.1 M, aqueous) was added. Similarly, to go from the doubly to singly deprotonated **PBI‐A**, 1 equivalent of HCl (0.1 M, aqueous was added), changing the pH 9 solution to pH 6. The resulting gels showed a single yield point as with the previous gels (Figures S17 and S18, Supporting Information). The strain at which the gels break (Table S7, Supporting Information) differs between the two different pH 6 samples (G′ value of 2800 for the switched to pH 6* (referred to as Gel 4), compared to 150 Pa for Gel 2), (Figure 5a). The gels formed from the solutions at the higher pH (Figures 3d and S18, Supporting Information) showed some similarities in stiffness (G′ values of 623 Pa and 540 Pa for the Gel 1 and from pH 9* (Referred to as Gel 3), respectively) despite the solutions being prepared differently. This can be ascribed to the increased solubility at high pH, meaning the structures are not “locked in”. Therefore, when the pH is lowered with GdL, the solutions follow the same kinetic pathway. With the pH 6*, these structures form as the pH decreases. When the equivalent of HCl is added this is different method used to lower the pH compared to GdL in gelation, which again differs to how the samples are prepared at pH 6, just using base. Therefore, the solutions do not pass through the same structures due to differences in kinetics, giving different gels.

The pH 9 solution and the pH 9* solutions showed very similar molecular packing (Figures 2a, S26, S2 and S28 Supporting Information), supporting the theory that no structures are “locked in” at the higher pH. Raising the pH results in the same structure despite the different ways in which the pH was changed, as there are limited possibilities available due to the solubility. However, the SANS from the pH 9* was able to be fit to a power law suggesting ill‐defined large structures are present (Figure S31 and Table S8, Supporting Information). Gel 3 also had very similar UV‐vis spectra (Figure S26, Supporting Information) and SANS showed that this gel now fits to a power law with a smaller value compared to a flexible elliptical cylinder with a power law for Gel 1 (Figure 3b and S32 and Tables S4 and S9, Supporting Information). The value suggests a porous structure but on too large a length scale to captured by SANS.

In comparison, there were differences in the absorption and emission spectra of the two differently prepared singly deprotonated solutions (Figures S25, S26, S27 and S28, Supporting Information). The 490 : 540 nm peak ratios were different (0.89 : 0.64 and 0.94 : 0.74 for the pH 6 and pH 6*, respectively), further suggesting differences in the starting structures present. Furthermore, pH 6* had a broader absorption, suggesting these solutions were more aggregated. SANS revealed that these samples now fitted to a power law of 3.4 (Figure S33 and 3b and Tables S10 and S3, Supporting Information). These structural differences resulted in differences in the UV‐vis spectra of the corresponding gels (Figure 5 (b) and Figure S25, Supporting Information). SANS from Gel 4 still fit to an elliptical cylinder with a power law, as for Gel 2. The fibres in Gel 4 were much more tape like with a smaller Kuhn length than seen for Gel 2. This difference means that the fibres are more flexible, giving a Gel 4 a larger bulk gel stiffness (Figure S34 and Table S11, Supporting Information). This difference could account for the significant difference in mechanical properties.

We hypothesised this property dependence on starting pH is not only for **PBI‐A**, and that gelators with two or more ionisable groups would also be sensitive to starting pH. We tested PBI−V, PBI‐Y^[10b]^ and PBI‐L^[14]^ using the same method as for **PBI‐A** (Figure S35, Supporting Information). In solution all PBIs showed different packing in the UV‐vis absorption spectroscopy at pH 9 and pH 6 (Figures S36–S36, Supporting Information). We also found that in all cases the gels formed from solution at pH 9 were considerably stiffer than those formed from pH 6, as with **PBI‐A** (Figures S37–S47, Supporting Information). Again, the absorption spectra of the gels showed that these were differently aggregated depending on which pH gelation was started from (Figures S48–S50). This demonstrates the importance in starting pH when carrying out gelation for multiple systems.

## Conclusion

All these data show the importance of the sample history of the solutions. We can access three different gels with widely different mechanical properties simply by changing the pH at which we start gelling, and how we get to the pre‐assembled structures in solution. The differences in the gels have huge consequences on the suitability of the gels for certain applications.

**Figure 6:**
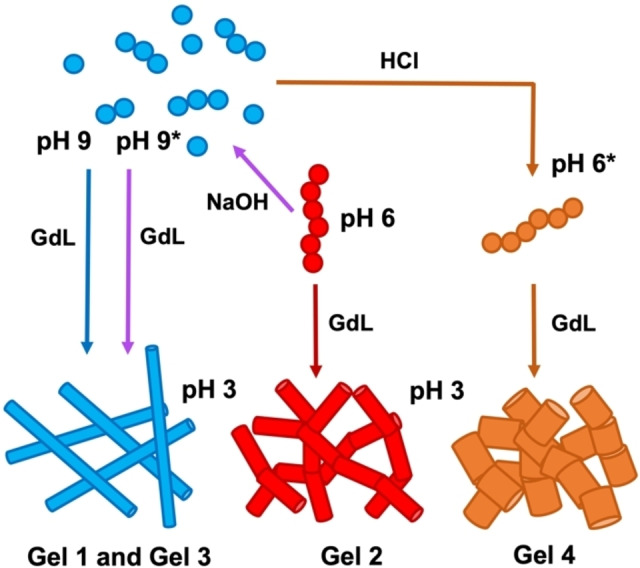
Cartoon illustrating how the different pathways to form aggregates and gels directly influence the network and gel properties.

At pH 9, the PBI molecules are more soluble and form less defined structures in solution and so at this pH the gelation kinetics are likely to be the same and form the same gel (Figure 6). Upon addition of GdL, pH 9 solutions form gels with more rigid cylindrical fibres. At pH 6, the molecules are already assembled and lowering the pH forms more flexible gel networks and fibres. We also find that you cannot change between the different aggregated states from pH 9 to pH 6 if these self‐assembled structures are already pre‐formed, which again allows us to form gels with different properties. For example, two gels here both formed from pH 6 aggregated states had more than an order of magnitude in difference in their stiffness, and thicker, flatter fibres. Such results will allow us to tailor our systems to meet the requirements of the chosen application whilst also highlighting the importance of sample preparation before triggering gelation.

## Experimental Section

Full experimental procedures and protocols can be found in the Supporting Information.

Preparation of PBI−A solutions: All solutions were prepared to a concentration of 5 mg/mL by dissolving PBI−A in distilled water and adding either two molar equivalents or 1 molar equivalent of sodium hydroxide (0.1 M, aqueous). To adjust a solution from 1 molar equivalent to 2 molar equivalents, 1 molar equivalent of NaOH (0.1 M, aqueous) was added and the sample was mixed for 10 min. To adjust a solution from 2 molar equivalents to 1 molar equivalent, 1 molar equivalent of HCl (0.1 M, aqueous) was added and the sample was mixed for 10 min.

Preparation of PBI−A hydrogels: A pH switch method was used to form the hydrogels. Solutions were prepared as above. 2 mL of solution was then transferred to a 7 mL Sterilin vial containing a pre‐weighed amount of glucono‐δ‐lactone (GdL) and gently shaken three times. The sample was then left to stand overnight to allow gelation to occur. For solutions with a starting pH of 6, 7.5 mg/mL of GdL was used and 10 mg/mL of GdL was used for solutions with a starting pH of 9. A simple inversion test was performed to indicate whether gel formation had been successful. If the sample was stable to inversion, then rheological measurements could be taken.

Rheological measurements: Dynamic rheological and viscosity measurements were performed with an Anton Paar Physica MCR101 and MCR301 rheometer. A cup‐and‐vane measuring system were used for strain and frequency sweeps; a cone‐and‐plate measuring system for viscosity measurements; and a parallel plate measurement system was used for time sweeps. For strain and frequency tests, 2 mL of gels were prepared in 7 mL Sterilin vials and left for 16 h at room temperature before measurements were taken. For viscosity measurements, PBI−A solutions were prepared as previously discussed. For time sweeps and gelling under constant shear, the gels were prepared in a vial and quickly transferred onto the bottom plate. The temperature was maintained at 25 °C during all measurements by using a water bath. All measurements were recorded in triplicate.

SANS: Solutions were prepared as described above in D2O and NaOD (0.1 M). SANS measurements were formed using the Larmor instrument (ISIS, Rutherford Appleton Laboratory, Didcot, UK) under experiment RB2210011, using a wavelength band of 0.9 to 13 Å to access a q range of 0.004 to 0.7 Å‐1. Solutions and gels were measured in 2 mm path length UV spectrophotomer grade quartz cuvettes (Hellma). They were placed in a temperature‐controlled sample rack during the measurements. Gels formed using GdL were prepared in a Sterilin vial and quickly transferred to the cuvettes, before being placed on the rack.

UV‐vis absorption spectroscopy: Solution UV‐vis absorption data were obtained with an Agilent Cary 60 UV‐vis spectrophotometer. The samples were prepared and transferred into a 0.1 mm quartz cuvette. Gels were made by the pH switch method as above but with an aliquot of the sample was then transferred to a 0.1 mm quartz cuvette, and tightly wrapped in parafilm to prevent the gel from drying out. Samples were then left overnight to gel.

Fluorescence spectroscopy: Fluorescence spectra were collected using an Agilent Cary Eclipse fluorescence spectrophotometer. The samples were prepared and transferred into 10.0 mm quartz cuvettes. Emissions and excitation spectra were recorded with slit widths of 10 nm at a scan rate of 600 nm/min. Emission spectra were collected between 700 and 200 nm, exciting at 365 nm. Spectra were recorded at a concentration of 0.05 mg/mL.

## Supporting Information

Additional references cited within the Supporting Information.[^13^, ^25^]

## Conflict of interest

The authors declare no conflict of interest.

1

## Supporting information

As a service to our authors and readers, this journal provides supporting information supplied by the authors. Such materials are peer reviewed and may be re‐organized for online delivery, but are not copy‐edited or typeset. Technical support issues arising from supporting information (other than missing files) should be addressed to the authors.

Supporting Information

## Data Availability

The data that support the findings of this study are available in the supplementary material of this article.
