# Supplementary material for: Using Solution History to Control Hydrogel Properties of a Perylene Bisimide
Source: Chemistry. 2023 May 10;29(37):e202301042. doi: 10.1002/chem.202301042 (PMC10947066; doi:10.1002/chem.202301042)
Supplement: Supplementary file 1 — Supporting Information [file CHEM-29-0-s001.pdf]

Chemistry–A European Journal

Supporting Information

Using Solution History to Control Hydrogel Properties of a Perylene Bisimide

Rebecca E. Ginesi, Nicholas R. Murray, Robert M. Dalgliesh, James Doutch, and Emily R. Draper*

Table of Contents

1. Experimental Procedures and Protocols S1

2. Supplementary Figures and Tables
- Solution pH 6 and pH 9 data (viscosity, UV-vis absorption, SANS) S2
- Gel 1 and gel 2 data (UV-vis absorption, rheology, images, scattering, kinetics) S4
- pH 6*, pH 9*, gel 3 and gel 4 data (rheology, SANS, UV-vis absorption etc.) S11
- Other gel data (PBI-Y, PBI-L and PBI-V) S21

3. References S29

1. Experimental Procedures and Protocols

Materials: PBI-A, PBI-L, **PBI-V** and **PBI-Y** were synthesized as reported previously.[1] Perylene-3,4-9,10-tetracarboxylic dianhydride (PTCDA), L-alanine, L-leucine, L-valine, L-tyrosine and imidazole were purchased from Sigma-Aldrich. All commercial reagents were used as received. Distilled water was used throughout. The NaOD was purchased from Sigma-Aldrich as a 40 wt% solution in D_2O and diluted with D_2O to provide a 0.1 M solution.

Preparation of PBI solutions: All solutions were prepared to a concentration of 5 mg/mL by dissolving the chosen PBI in distilled water and adding either two molar equivalents or 1 molar equivalent of sodium hydroxide (0.1 M, aqueous). The solutions were stirred until all the gelator had dissolved.

Preparation of pH-switched solutions: To adjust a solution from 1 molar equivalent to 2 molar equivalents, 1 molar equivalent of NaOH (0.1 M, aqueous) was added and the sample was mixed for 10 minutes. To adjust a solution from 2 molar equivalents to 1 molar equivalent, 1 molar equivalent of HCl (0.1 M, aqueous) was added and the sample was mixed for 10 minutes.

Preparation of PBI hydrogels: A pH switch method was used to form the hydrogels. Solutions were prepared as above. 2 mL of solution was then transferred to a 7 mL Sterilin vial containing a pre-weighed amount of glucono-δ-lactone (GdL) (Tables S1 and S12) and gently shaken three times. The sample was then left to stand overnight to allow gelation to occur. A simple inversion test was performed to indicate whether gel formation had been successful. If the sample was stable to inversion, then rheological measurements could be taken.

pH measurements: pH measurements were recorded on an FC200 pH probe (HANNA instruments) with a 6 mm x 10 mm conical tip at 25°C. The stated accuracy of the pH measurement is ± 0.1.

UV-vis absorption spectroscopy: Solution UV-vis absorption data were obtained with an Agilent Cary 60 UV-vis spectrophotometer. The samples were prepared and transferred into a 0.1 mm quartz cuvette. Gels were made by the pH switch method, by lowering the pH with the appropriate amount of GdL. The GdL was added to a 2 mL solution in a 7 mL Sterilin vial and gently shaken three times. An aliquot of the sample was then transferred to a 0.1 mm cuvette, and tightly wrapped in parafilm to prevent the gel from drying out. Samples were then left overnight to gel.

Fluorescence spectroscopy: Fluorescence spectra were collected using an Agilent Cary Eclipse fluorescence spectrophotometer. The samples were prepared and transferred into 10.0 mm quartz cuvettes. Emissions and excitation spectra were recorded with slit widths of 10 nm at a scan rate of 600 nm/min. Emission spectra were collected between 700 and 200 nm, exciting at 365 nm. Spectra were recorded at a concentration of 0.05 mg/mL.

Rheological measurements: Dynamic rheological and viscosity measurements were performed with an Anton Paar Physica MCR101 and MCR301 rheometer. A cup-and-vane measuring system were used for strain and frequency sweeps; a cone-and-plate measuring system for viscosity measurements; and a parallel plate measurement system was used for time sweeps. For strain and frequency tests, 2 mL of gels were prepared in 7 mL Sterilin vials and left for 16 hours at room temperature before measurements were taken. For viscosity measurements, **PBI-A** solutions were prepared as previously discussed. For time sweeps and gelling under constant shear, the gels were prepared in a vial and quickly transferred onto the bottom plate. The temperature was maintained at 25°C during all measurements by using a water bath. All measurements were recorded in triplicate.

Strain sweeps: Strain sweeps were performed over a range of 0.1% to 1000%, with a frequency of 10 rad/s. The critical strain was quoted at the point where the storage modulus (G') began to become non-linear and ultimately crossed over the loss modulus (G"), consequently causing the gel to break down.

Frequency sweeps: Frequency sweeps were performed from 1 rad/s to 100 rad/s under a strain of 0.1%. The shear modulus (G' and G") were quoted at 10 rad/s. The measurements were performed within the viscoelastic region where G' and G" were independent of strain amplitude.

Viscosity measurements: Viscosity measurements were performed with a 75 mm cone (angle = 1.000°) and a plate gap of 0.1 mm. 2 mL solutions were poured onto the plate for the measurement. The viscosity was measured under the rotation shear rate from 1 to 100 s^{-1}.

Time sweeps: Time sweeps were performed with a 50 mm sandblasted plate with a plate gap of 0.8 mm. Tests were performed at an angular frequency of 10 rad/s and with a strain of 0.1%. Mineral oil was added to the top plate to prevent the sample from drying out.

SANS: Solutions were prepared as described above in D_2O and NaOD (0.1 M). SANS measurements were formed using the Larmor instrument (ISIS, Rutherford Appleton Laboratory, Didcot, UK) under experiment RB2210011, using a wavelength band of 0.9 to 13 Ang to access a q range of 0.004 to 0.7 Ang^{-1}. Solutions and gels were measured in 2 mm path length UV spectrophotomer grade quartz cuvettes (Hellma). They were placed in a temperature-controlled sample rack during the measurements. Gels formed using GdL were prepared in a Sterilin vial and quickly transferred to the cuvettes, before being placed on the rack.

The data was reduced to 1D scattering curves of intensity vs Q. using the facility provided software. The electronic background was subtracted, the full detector images for all data were normalised and scattering from the empty cell was subtracted. The scattering from D_2O was also measured and subtracted from the data using the Mantid software package installed inside the ISIS virtual machines, IDAaaS.[2] The instrument-independent data were then fitted to the models discussed in the text using the SasView software package (version 5.0.4).[3] The scattering length density of each material was calculated using the National Institute of Standard and Technology's neutron activation and scattering calculator.[4] The SLD of D_2O was calculated to be 6.393 x 10^{-6} $Å^{-2}$ and the SLD of PBI-A was calculated to be 3.445 x 10^{-6} $Å^{-2}$. All data fitted best to a cylindrical model combined with a power law. The specific model is described in the figure captions and tabulated parameters can be found in Tables S3-S5. The best fit was determined as the one which fit well to the data and had the lowest $\chi 2$ value.

"Apparent" p*K*a titrations: An FC200 pH probe (HANNA instruments) with a 6 mm x 10 mm conical tip was used for pK_a titrations. The "apparent" pK_a values of **PBI-A** were determined by the addition of aliquots of 0.1 M aqueous hydrochloric acid. The pH was recorded after each aliquot addition once a stable pH was maintained. To prevent gel formation, the solutions were gently stirred using a stirrer bar. For pH titrations using GdL, the correct concentration of GdL was added to a 2 mL sample in a Sterilin vial and recorded overnight. The temperature was maintained at 25°C by using a water bath. The plateaus of the pH are indicative of the "apparent" pK_a values for the gelator. "Apparent" here refers to a change in aggregation which causes a plateau in the pH data which is brought about by the protonation of the COOH groups. pK_a of COOHs range from 5-2.

^{1}H NMR Kinetics: ^{1}H NMR spectra were measured using a Bruker 500 MHz spectrometer operating at 25°C. Spectra were analyzed using Bruker Topspin 4.1.0. Solutions were prepared as described above in D_2O (as opposed to H_2O) and 0.1 M NaOD (as opposed to NaOH). MeOH (2 μL) was then added as an internal standard. For the time-zero measurement (t_0), a ^{1}H NMR spectrum of 1000 μL of the stock solution was recorded. 2 mL of the gelator solution was then mixed with GdL in a Sterilin vial and 1000 μL was directly loaded into an NMR tube to gel. Due to experimental limitation, there was a 10-minute delay between the addition of the sample to the spectrometer and the first measurement. During the gelation period, spectra were recorded every 5 minutes until the protons on the methyl group of the **PBI-A** were no longer detectable. The methyl protons of **PBI-A** were then integrated and compared to the integration value for the **PBI-**A methyl peak in the t_0 spectrum. When assembled, PBIs become NMR invisible as they are unable to diffuse,[5] which can be used to calculate the percentage unassembled. From this, the percentage assembled was calculated and plotted against time.

2. Supplementary Figures and Tables

Table S1. Concentration of glucono-δ-lactone required to form hydrogels from **PBI-A** solutions with different equivalents of 0.1 M NaOH (aq.) with a final pH of approximately 3.2. pH data shown are averaged data for triplicate samples, with error being calculated using standard deviation.

**Gel**	GdL Concentration (mg/mL)	Average pH
1	7.5	3.22 ± 0.010
2	10	3.27 ± 0.005
3	10	3.20 ± 0.020
4	7.5	3.19 ± 0.005

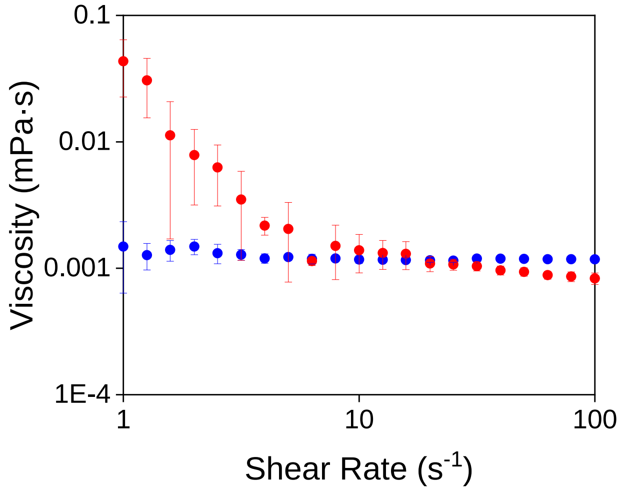

Figure S1. Viscosity measurements of **PBI-A** solutions at pH 6 (red) and pH 9 (blue). Data shown are averaged data for triplicate runs of the samples, with error bars being calculated using standard deviation.

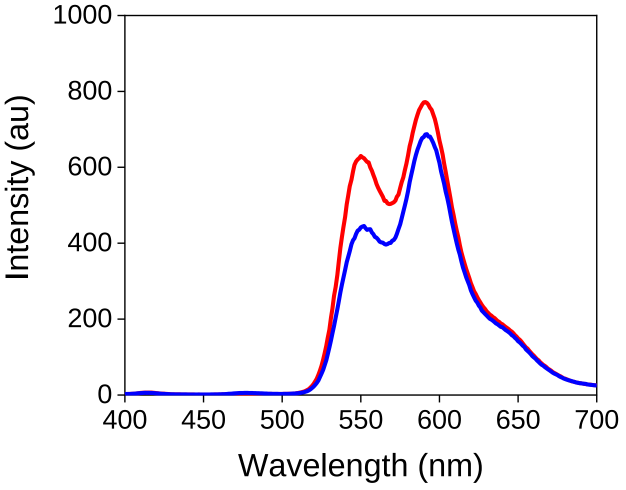

Figure S2. Fluorescence spectra of **PBI-A** solutions at pH 6 (red) and pH 9 (blue). Fluorescence spectra were collected at an excitation of 365 nm and at a gelator concentration of 0.05 mg/mL.

Table S2. Concentration of glucono-δ-lactone required to form hydrogels from **PBI-A** solutions with different equivalents of 0.1 M NaOD (aq) with a final pD of approximately 3.6, and therefore, a final pH of 3.2. pH.

**Gel**	GdL Concentration (mg/mL)	Average pD	pH (pH = pD – 0.4)
1	7.5	3.64	3.24 ± 0.025
2	10	3.60	3.20 ± 0.005

Table S3. Parameters of SANS model fit for a **PBI-A** solution at pD 6.

**Flexible Elliptical Cylinder + Power Law**	Value	Error
Background (cm^{-1})	0.007	
Cylinder Scale	1.8153 $x10^{-4}$	4.3214 x 10^{-6}
Length (Å)	2483.8	502
Kuhn Length (Å)	179	5.2
Radius (Å)	42.71	0.53
Axis Ratio	2.29	0.04
Power Law Scale	3.2971 $x10^{-6}$	3.1231 $x10^{-7}$
Power Law	2.80	0.02
χ^2	2.5789	

Table S4. Parameters of SANS model fit for the gel made from a **PBI-A** solution starting at pD 9 (Gel 1).

**Elliptical Cylinder + Power Law**	Value	Error
Background (cm^{-1})	0.007	
Cylinder Scale	7.5974 $x10^{-4}$	8.7217 x 10^{-6}
Length (Å)	327.7	4.4
Radius (Å)	60.9	0.3
Axis Ratio	1.9	0.02
Power Law Scale	4.8562 $x10^{-6}$	2.8229 $x10^{-7}$
Power Law	2.90	0.01
χ^2	3.5974	

Table S5. Parameters of SANS model fit for the gel made from a **PBI-A** solution starting at pD 6 (Gel 2).

**Flexible Elliptical Cylinder + Power Law**	Value	Error
Background (cm^{-1})	0.007	
Cylinder Scale	2.2163 x10^{-4}	2.0519 x 10^{-6}
Length (Å)	2483.8	502
Kuhn Length (Å)	112.6	8.4
Radius (Å)	34.5	0.8
Axis Ratio	2.79	0.05
Power Law Scale	6.339 x10^{-6}	5.077 x10^{-7}
Power Law	2.84	0.02
χ^2	3.4305	

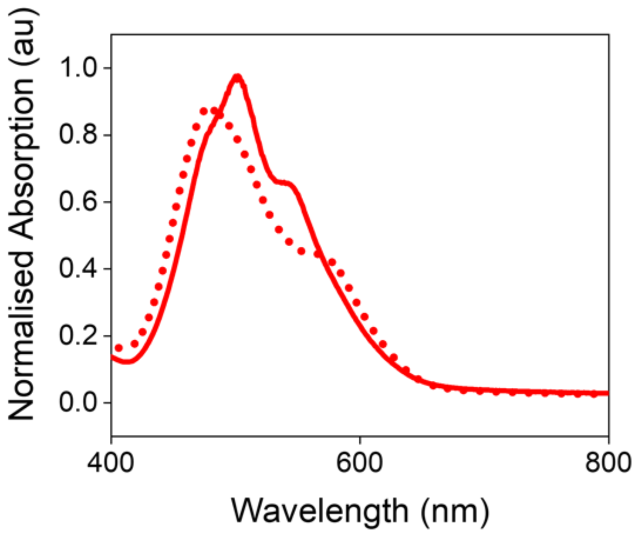

Figure S3. UV-vis absorbance spectra of solutions (solid line) at pD 6 and their corresponding gels at pH 3.2 (dotted line).

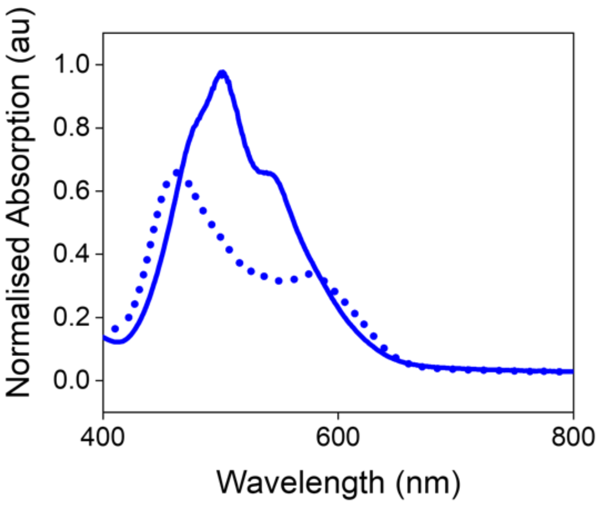

Figure S4. UV-vis absorption spectra of solutions (solid line) at pD 9 and their corresponding gels at pH 3.2 (dotted line).

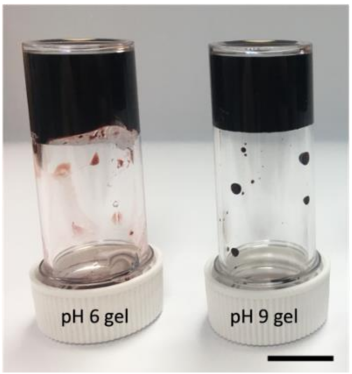

Figure S5. Photograph of gels at pH 3.2 formed from **PBI-A** solutions starting at pH 6 (left) and pH 9 (right). Scale bar represents 1 cm.

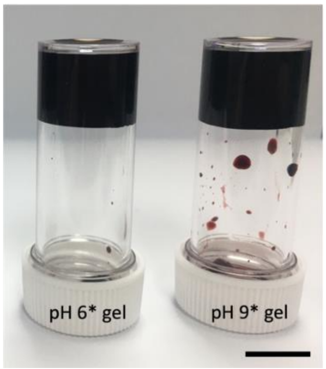

Figure S6. Photograph of gels at pH 3.2 formed from **PBI-A** solutions starting at pH 6* (left) and pH 9* (right). Scale bar represents 1 cm.

Table S6. Tabulated rheological properties taken from an average of three rheological strain sweep measurements for gels formed from **PBI-A** solutions starting at pH 6 (Gel 2) and pH 9 (Gel 1). G′ and G″ are quoted at a strain of 0.1%.

**Gel**	G′ (Pa)	G″ (Pa)	Yield Point (%)	Flow Point (%)
2	147 ± 5	9 ±1	10	80
1	623 ± 27	111 ± 12	2.5	40

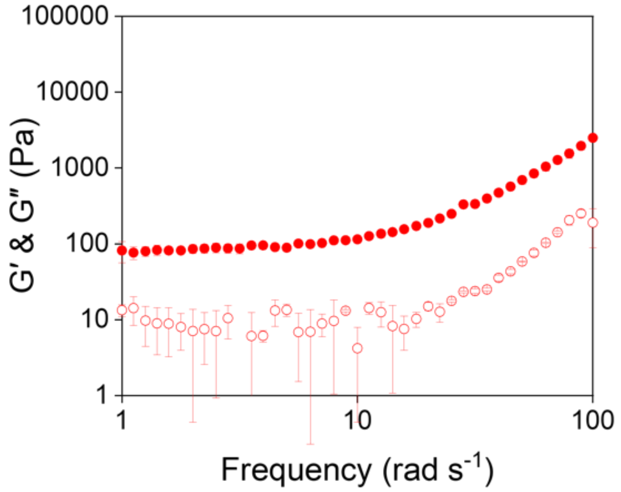

Figure S7. Frequency sweeps of a gel formed from solutions of **PBI-A** starting at pH 6 (Gel 2). Closed circles represent G′ and open circles represent G″. Frequency sweeps were performed at 0.1% strain at 25°C. Data shown is averaged data for triplicate runs of the samples, with the error bars being calculated using standard deviation.

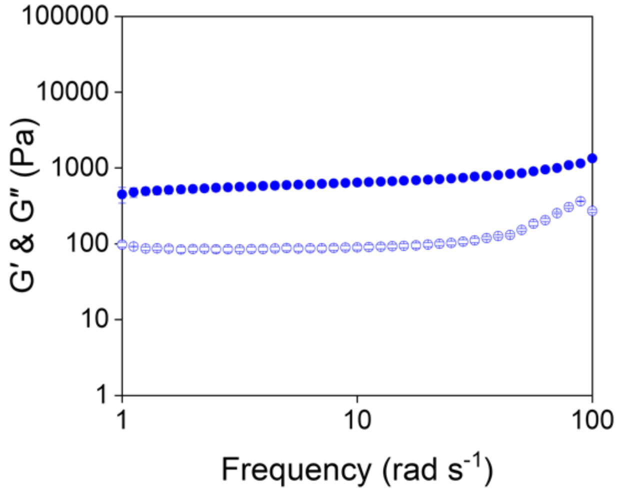

Figure S8. Frequency sweeps of a gel formed from solutions of **PBI-A** starting at pH 9 (Gel 1). Closed circles represent G′ and open circles represent G″. Frequency sweeps were performed at 0.1% strain at 25°C. Data shown is averaged data for triplicate runs of the samples, with the error bars being calculated using standard deviation.

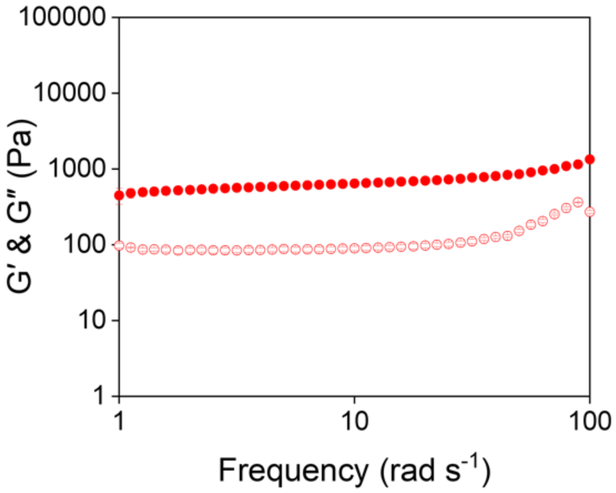

Figure S9. Frequency sweep of a gel formed from solutions of **PBI-A** starting at pD 6 (Gel 2). Closed circles represent G′ and open circles represent G″. Frequency sweeps were performed at a frequency of 0.1% strain at 25°C. Data shown is averaged data for triplicate runs of the samples, with the error bars being calculated using standard deviation.

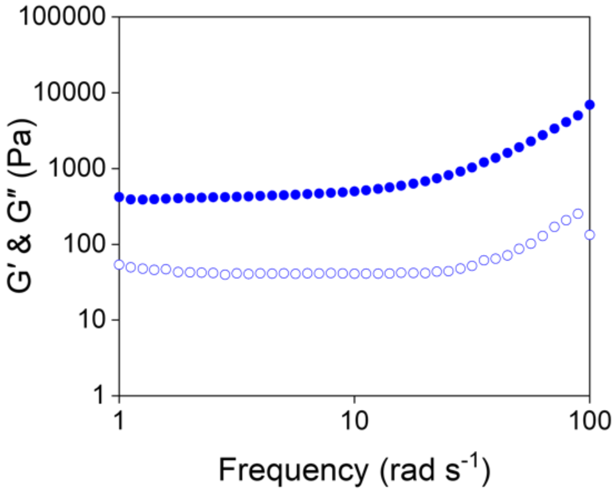

Figure S10. Frequency sweep of a gel formed from solutions of **PBI-A** starting at pD 9 (Gel 1). Closed circles represent G′ and open circles represent G″. Frequency sweeps were performed at a frequency of 0.1% strain at 25°C. Data shown is averaged data for triplicate runs of the samples, with the error bars being calculated using standard deviation.

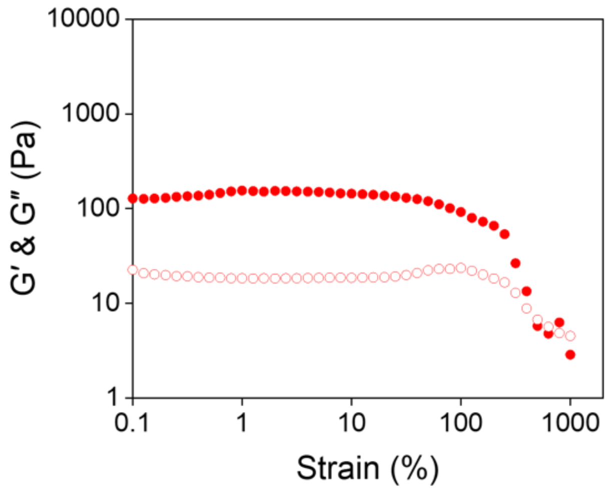

Figure S11. Strain sweep of a gel formed from solutions of **PBI-A** starting at pD 6 (Gel 2). Closed circles represent G′ and open circles represent G″. Strain sweeps were performed at a frequency of 10 rad s^{-1} at 25°C. Data shown is averaged data for triplicate runs of the samples, with the error bars being calculated using standard deviation.

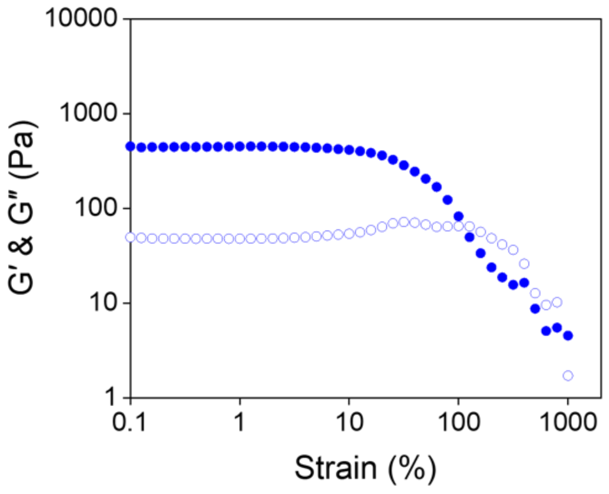

Figure S12 Strain sweep of a gel formed from solutions of **PBI-A** starting at pD 9 (Gel 1). Closed circles represent G′ and open circles represent G″. Strain sweeps were performed at a frequency of 10 rad s^{-1} at 25°C. Data shown is averaged data for triplicate runs of the samples, with the error bars being calculated using standard deviation.

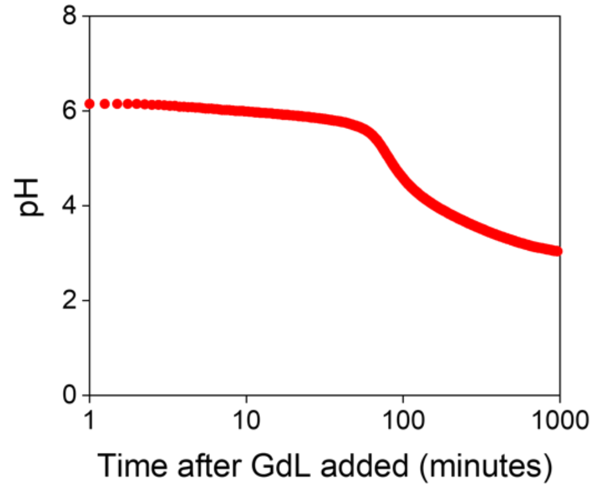

Figure S13. Change in pH upon addition of GdL to a solution of **PBI-A** starting at pD 6. Measurements were performed at 25°C.

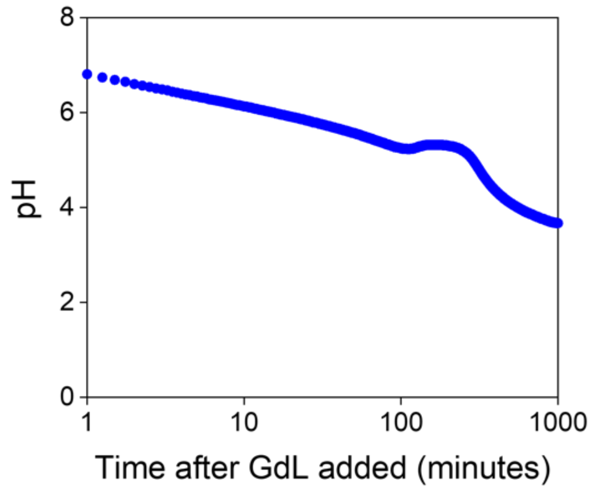

Figure S14. Change in pH upon addition of GdL to a solution of **PBI-A** starting at pD 9. Measurements were performed at 25°C.

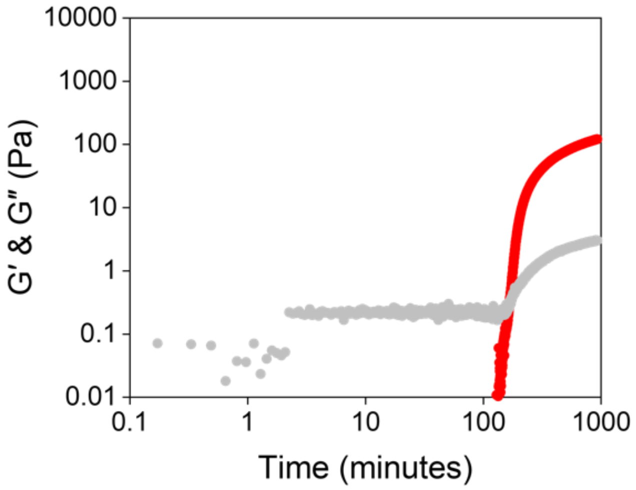

Figure S15. Rheological time-sweep measuring the development of G′ (red) and G″ (grey) with time of **PBI-A** solutions starting at pD 6. Measurements were performed under a strain of 0.1%, frequency of 10 rad s^{-1} and at 25°C.

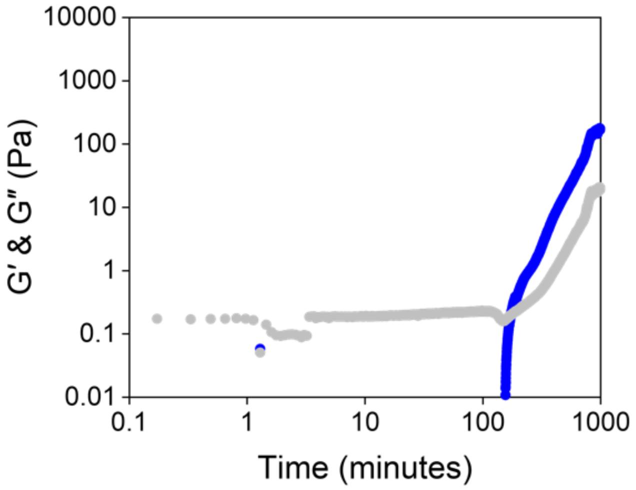

Figure S16. Rheological time-sweep measuring the development of G′ (blue) and G″ (grey) with time of **PBI-A** solutions starting at pD 9. Measurements were performed under a strain of 0.1%, frequency of 10 rad s^{-1} and at 25°C.

Table S7. Tabulated rheological properties taken from an average of three rheological strain sweep measurements for gels formed from **PBI-A** solutions at pH 6* (Gel 4) and pH 9* (Gel 3). G′ and G″ are quoted at a strain of 0.1%.

**Gel**	G′ (Pa)	G″ (Pa)	Yield Point (%)	Flow Point (%)
3	540 ± 74	91 ± 11	4	20
4	2850 + 172	468 ± 39	5	40

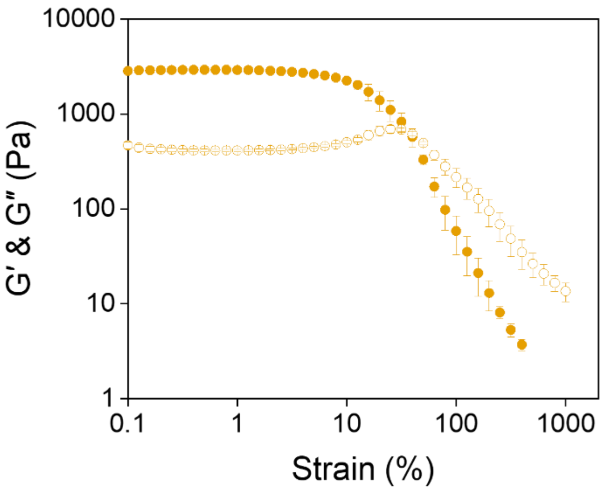

Figure S17. Strain sweep of a gel formed from solutions of **PBI-A** starting at pH 6* (Gel 4). Closed circles represent G′ and open circles represent G″. Strain sweeps were performed at a frequency of 10 rad s^{-1} at 25°C. Data shown is averaged data for triplicate runs of the samples, with the error bars being calculated using standard deviation.

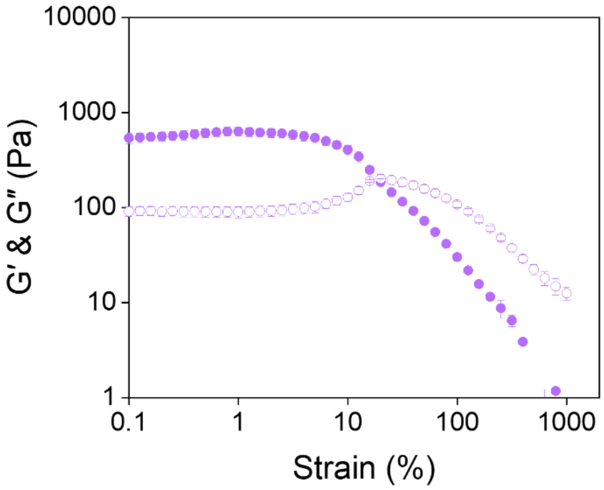

Figure S18. Strain sweep of a gel formed from solutions of **PBI-A** starting at pH 9* (Gel 3). Closed circles represent G′ and open circles represent G″. Strain sweeps were performed at a frequency of 10 rad s^{-1} at 25°C. Data shown is averaged data for triplicate runs of the samples, with the error bars being calculated using standard deviation.

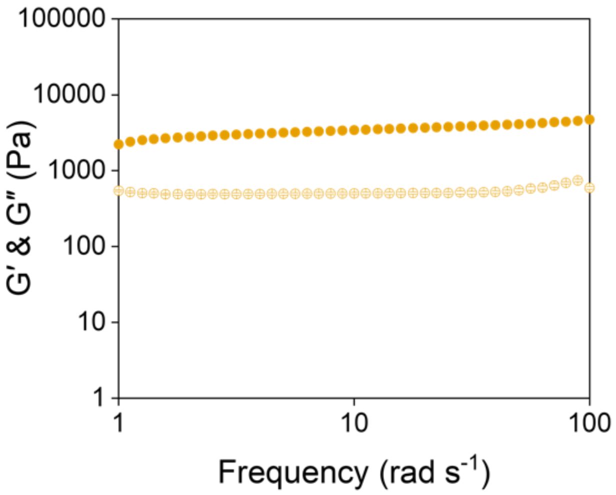

Figure S19. Frequency sweep of a gel formed from solutions of **PBI-A** starting at pH 6* (Gel 4). Closed circles represent G′ and open circles represent G″. Frequency sweeps were performed at a frequency of 0.1% strain at 25°C. Data shown is averaged data for triplicate runs of the samples, with the error bars being calculated using standard deviation.

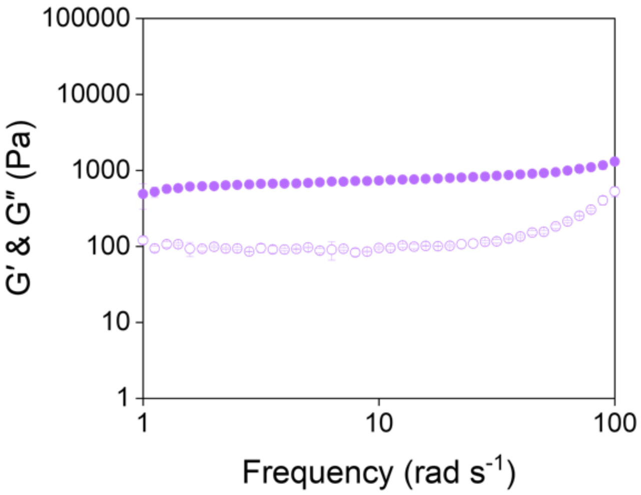

Figure S20. Frequency sweep of a gel formed from solutions of **PBI-A** starting at pH 9* (Gel 3). Closed circles represent G′ and open circles represent G″. Frequency sweeps were performed at a frequency of 0.1% strain at 25°C. Data shown is averaged data for triplicate runs of the samples, with the error bars being calculated using standard deviation.

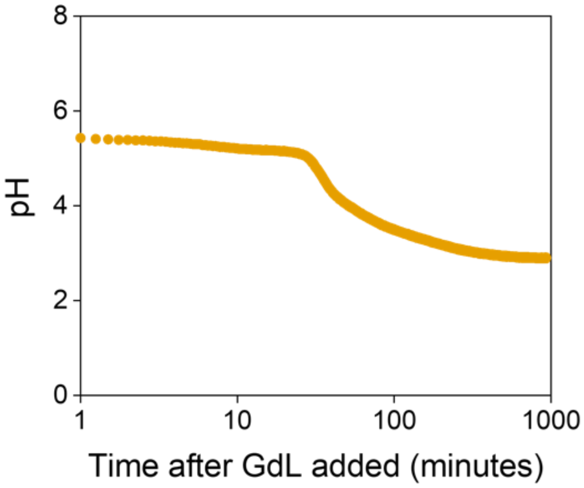

Figure S21. Change in pH upon addition of GdL to a solution of **PBI-A** at pH 6*. Measurements were performed at 25°C.

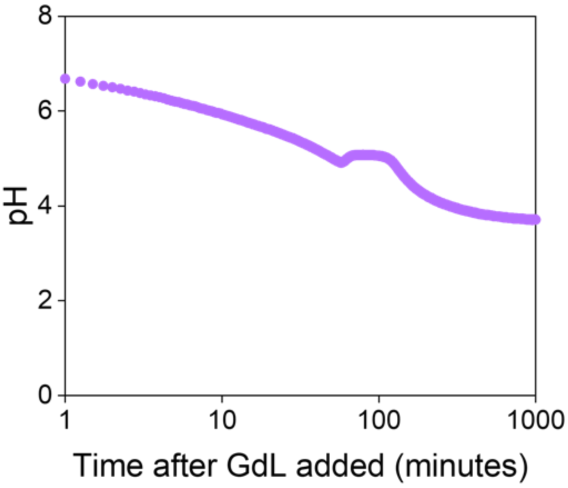

Figure S22. Change in pH upon addition of GdL to a solution of PBI-A at pH 9*. Measurements were performed at 25°C**.**

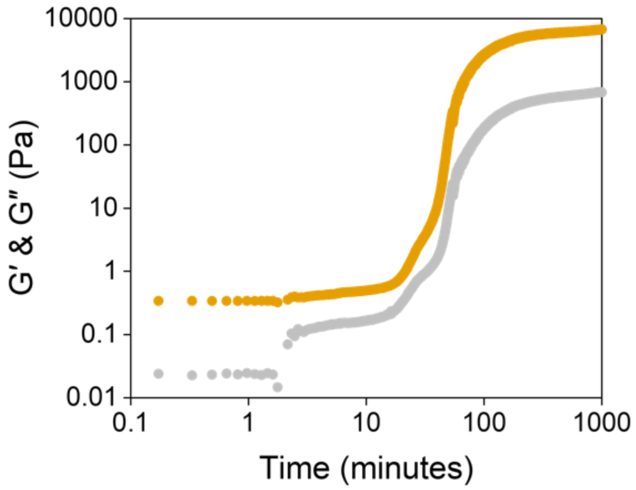

Figure S23. Rheological time-sweep measuring the development of G′ (orange) and G″ (grey) with time of **PBI-A** solutions at pH 6*. Measurements were performed under a strain of 0.1%, frequency of 10 rad s^{-1} and at 25°C.

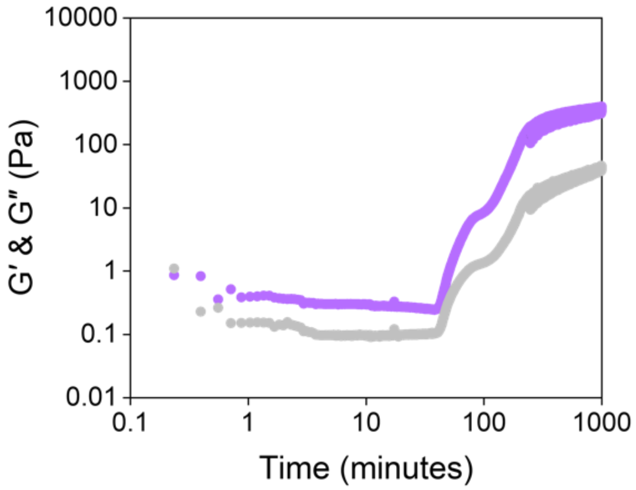

Figure S24. Rheological time-sweep measuring the development of G′ (purple) and G″ (grey) with time of **PBI-A** solutions at pH 9*. Measurements were performed under a strain of 0.1%, frequency of 10 rad s^{-1} and at 25°C.

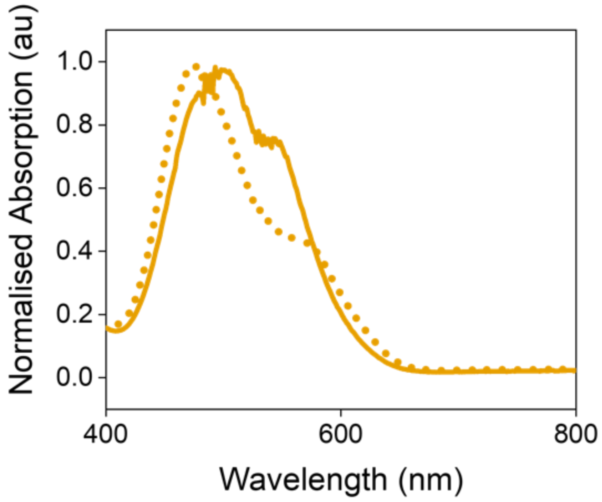

Figure S25. UV-vis absorbance spectra of **PBI-A** solutions at pH 6* and their corresponding gels (dotted line).

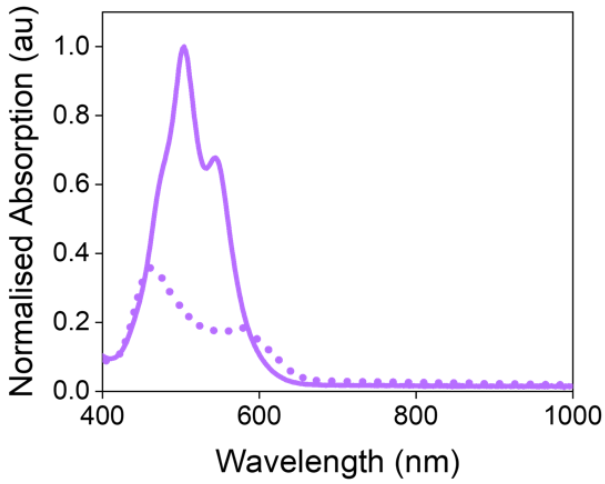

Figure S26. UV-vis absorbance spectra of **PBI-A** solutions (solid line) at pH 9* and their corresponding gels (dotted line).

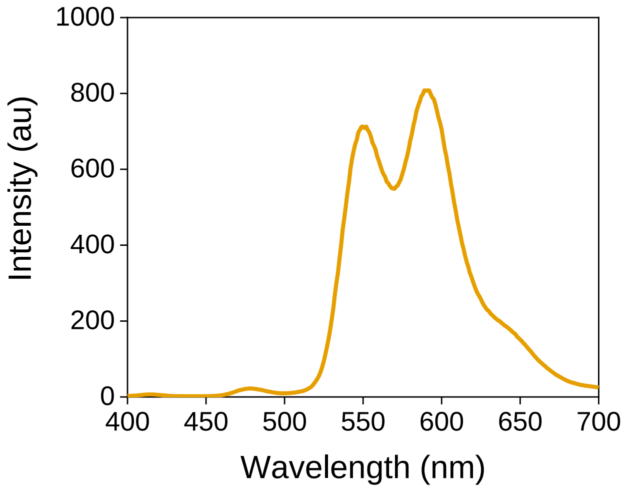

Figure S27. Fluorescence spectra of **PBI-A** solutions at pH 6*. Fluorescence spectra were collected at an excitation of 365 nm and at a gelator concentration of 0.05 mg/mL.

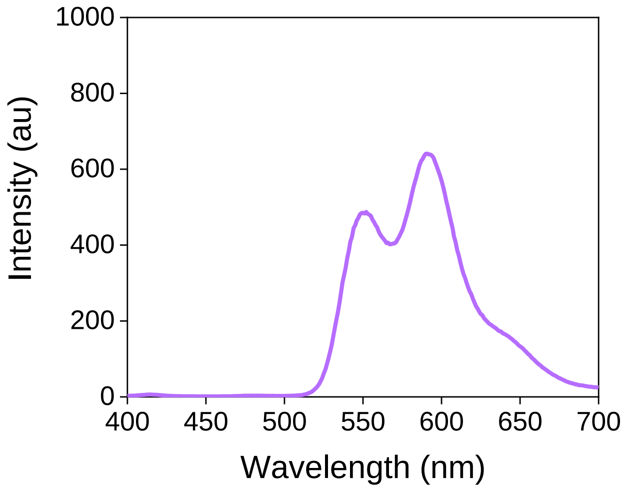

Figure S28. Fluorescence spectra of **PBI-A** solutions at pH 9*. Fluorescence spectra were collected at an excitation of 365 nm and at a gelator concentration of 0.05 mg/mL.

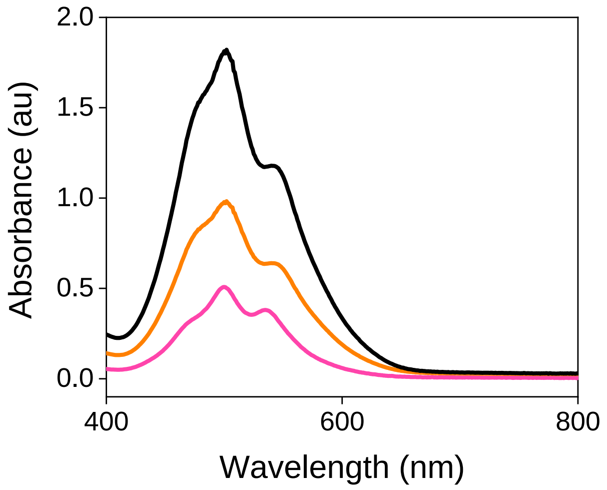

Figure S29. Absorption spectra of **PBI-A** solutions at pH 6 at 5 (black), 1 (orange) and 0.05 (pink) mg/mL.

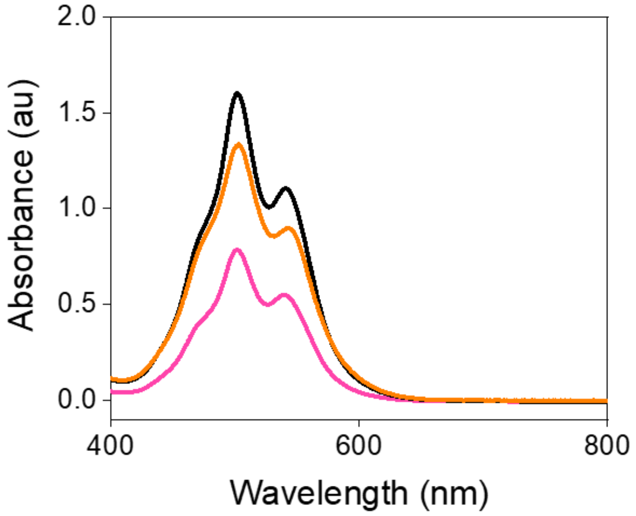

Figure S30. Absorption spectra of **PBI-A** solutions at pH 9 at 5 (black), 1 (orange) and 0.05 (pink) mg/mL.

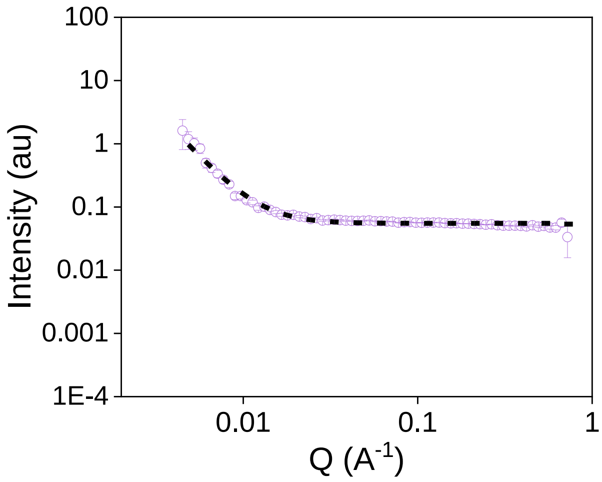

Figure S31. SANS of a **PBI-A** solution at pD 9* with fit in dashed line.

Table S8. Parameters of SANS model fit for a solution of **PBI-A** at pD 9*.

**Power Law**	Value	Error
Background (cm^{-1})	0.005	
Power Law Scale	1.1931 x10^{-7}	4.1349 x 10^{-9}
Power Law	2.90	0.03
χ^2	10.191	

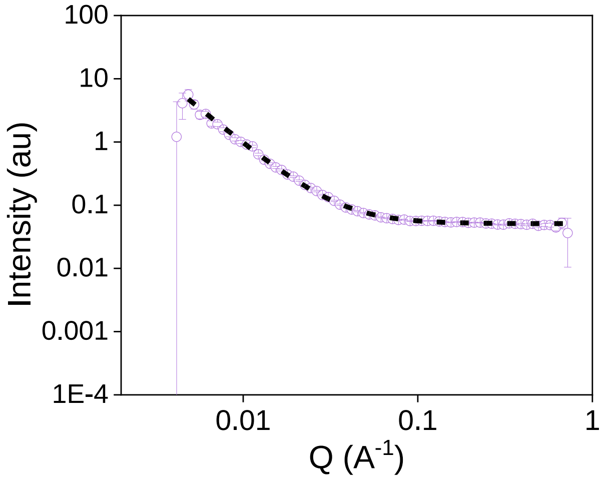

Figure S32. SANS of a gel formed from a **PBI-A** at pD 9* (Gel 3) with fit in dashed line.

Table S9. Parameters of SANS model fit for a gel formed from a solution of **PBI-A** at pD 9* (Gel 3).

**Power Law**	Value	Error
Background (cm^{-1})	0.005	
Power Law Scale	3.3286 x10^{-5}	2.1034 x 10^{-6}
Power Law	2.21	0.02
χ^2	2.5182	

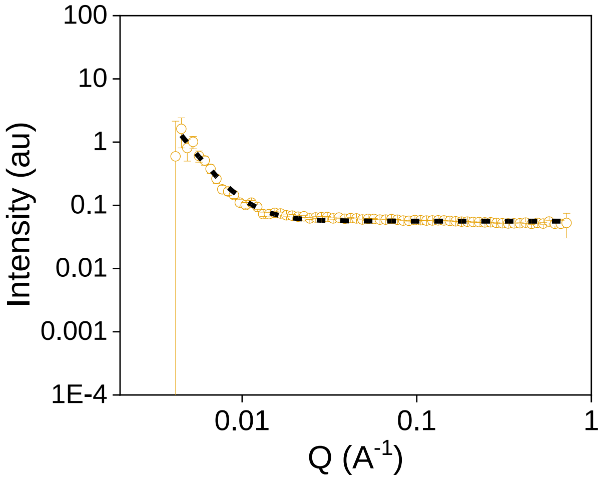

Figure S33. SANS of a **PBI-A** solution at pD 6* with the fit as a dashed line.

Table S10. Parameters of SANS model fit for a solution of **PBI-A** at pD 6*.

**Power Law**	Value	Error
Background (cm^{-1})	0.005	
Power Law Scale	8.3993 x10^{-9}	0.0001
Power Law	3.44	0.7
χ^2	10.443	

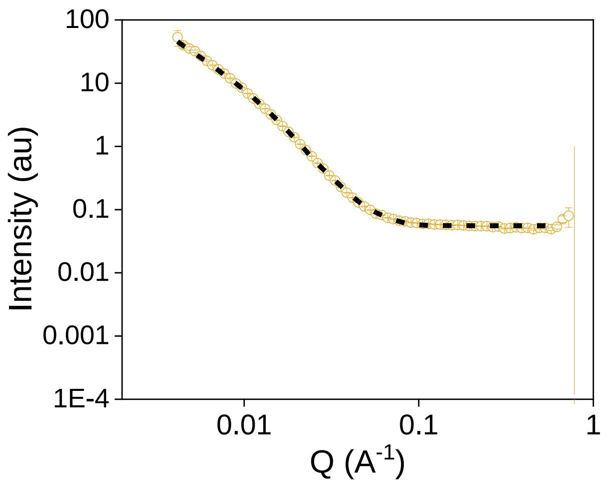

Figure S34. SANS of a gel formed from a **PBI-A** at pD 6* (Gel 4) with the fit in a dashed line.

Table S11. Parameters of SANS model fit for a gel formed from a solution of **PBI-A** at pD 6* (Gel 4).

**Flexible Elliptical Cylinder**	Value	Error
Background (cm^{-1})	0.005	
Cylinder Scale	0.00017459	6.159×10^{-6}
Length (Å)	1000	-
Kuhn Length (Å)	69.5	2.9
Radius (Å)	28.7	0.4
Axis Ratio	5.33	0.1
χ^2	9.3703	

Table S12. Concentration of glucono-δ-lactone required to form hydrogels from **PBI-L, PBI-V** and **PBI-Y** solutions with different equivalents of 0.1 M NaOH (aq.) pH data shown are averaged data for triplicate samples, with error being calculated using standard deviation.

**Gel**	GdL Concentration (mg/mL)	Average pH
**PBI-L** (1 eq)	5	3.44 ± 0.005
**PBI-L** (2 eq)	10	3.25 ± 0.005
**PBI-V** (1 eq)	5	3.65 ± 0.010
**PBI-V** (2 eq)	7.5	3.52 ± 0.010
**PBI-Y** (1 eq)	10	3.22 ± 0.010
**PBI-Y** (2 eq)	10	3.23 ± 0.010

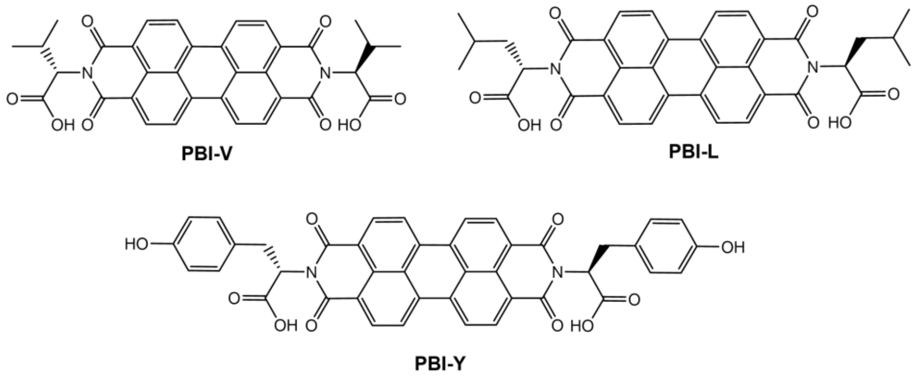

Figure S35. Chemical structures of **PBI-V**, **PBI-L** and **PBI-Y**

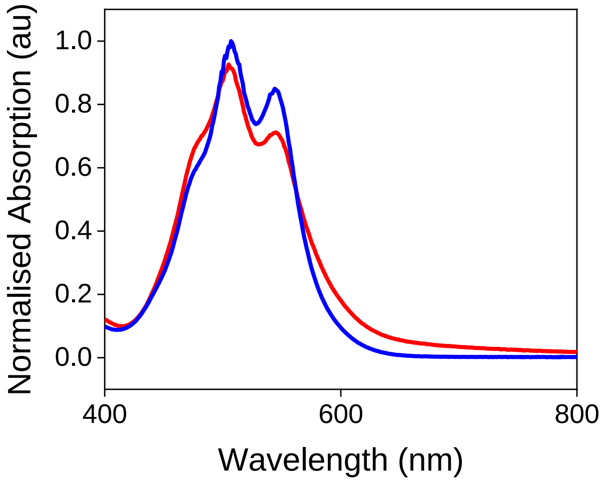

Figure S36. UV-vis absorption spectra of **PBI-L** solutions at pH 6 (red) and pH 9 (blue).

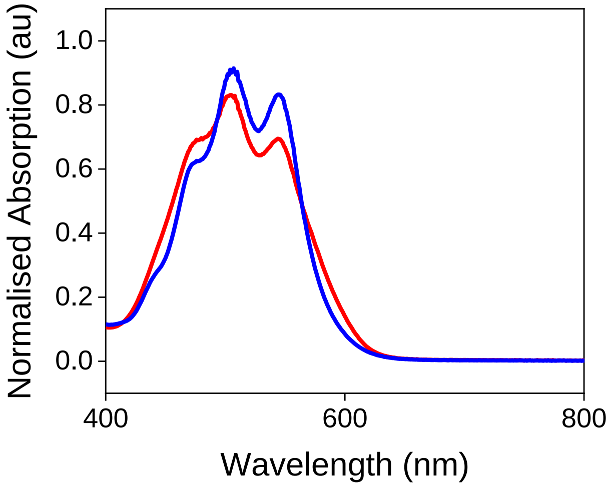

Figure S37. UV-vis absorption spectra of **PBI-V** solutions at pH 6 (red) and pH 9 (blue).

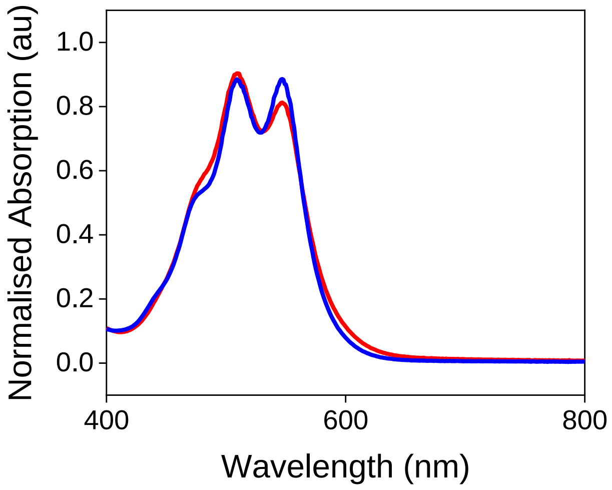

Figure S38. UV-vis absorption spectra of **PBI-Y** solutions at pH 6 (red) and pH 9 (blue).

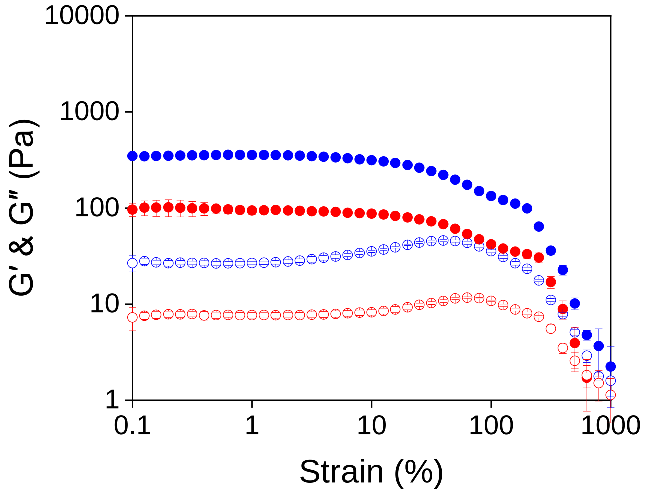

Figure S39. Strain sweeps of **PBI-L** gels formed from solutions starting at pH 6 (red) and pH 9 (blue). Closed circles represent G′ and open circles represent G″. Data shown are averaged data for triplicate runs, with error bars being calculated using standard deviation.

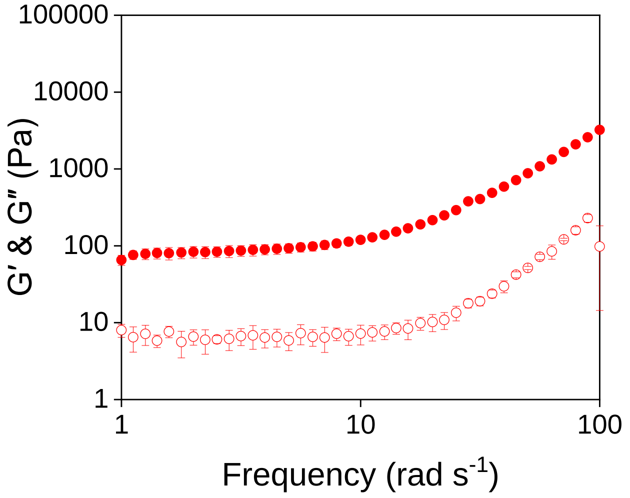

Figure S40. Frequency sweeps of a gel formed from solutions of **PBI-L** starting at pH 6. Closed circles represent G′ and open circles represent G″. Frequency sweeps were performed at 0.1% strain at 25°C. Data shown is averaged data for triplicate runs of the samples, with the error bars being calculated using standard deviation.

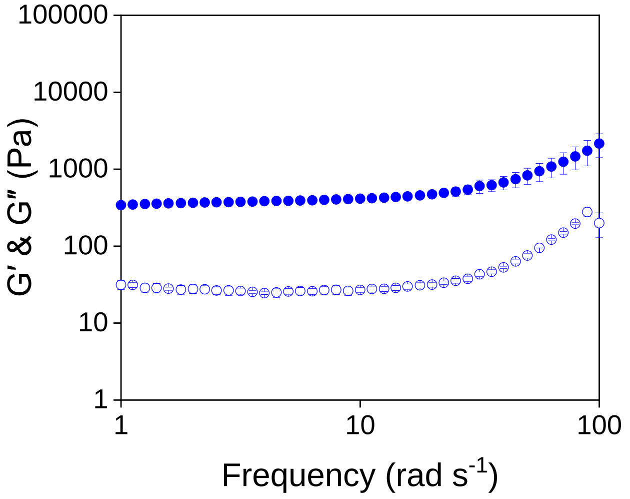

Figure S41. Frequency sweeps of a gel formed from solutions of **PBI-L** starting at pH 9. Closed circles represent G′ and open circles represent G″. Frequency sweeps were performed at 0.1% strain at 25°C. Data shown is averaged data for triplicate runs of the samples, with the error bars being calculated using standard deviation.

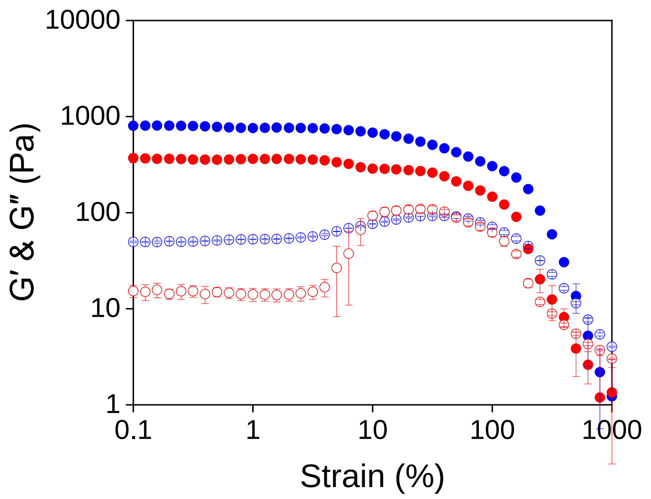

Figure S42. Strain sweeps of **PBI-V** gels formed from solutions starting at pH 6 (red) and pH 9 (blue). Closed circles represent G′ and open circles represent G″. Data shown are averaged data for triplicate runs, with error bars being calculated using standard deviation.

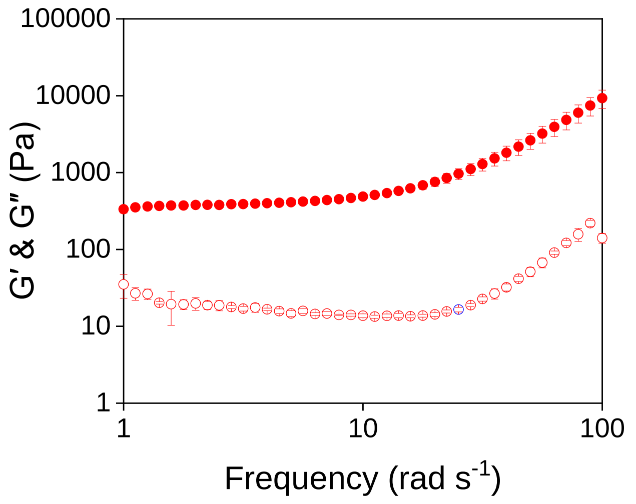

Figure S43. Frequency sweeps of a gel formed from solutions of **PBI-V** starting at pH 6. Closed circles represent G′ and open circles represent G″. Frequency sweeps were performed at 0.1% strain at 25°C. Data shown is averaged data for triplicate runs of the samples, with the error bars being calculated using standard deviation.

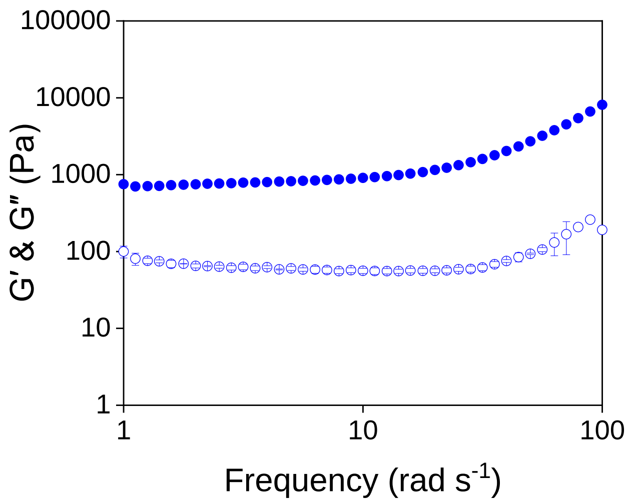

Figure S44. Frequency sweeps of a gel formed from solutions of **PBI-V** starting at pH 9. Closed circles represent G′ and open circles represent G″. Frequency sweeps were performed at 0.1% strain at 25°C. Data shown is averaged data for triplicate runs of the samples, with the error bars being calculated using standard deviation.

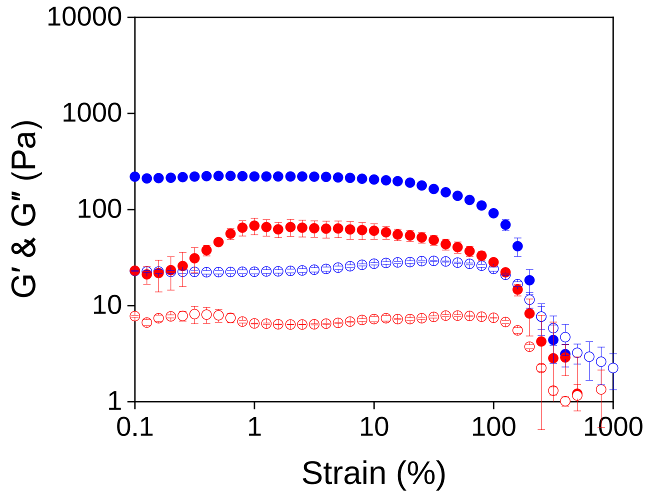

Figure S45. Strain sweeps of **PBI-Y** gels formed from solutions starting at pH 6 (red) and pH 9 (blue). Closed circles represent G′ and open circles represent G″. Data shown are averaged data for triplicate runs, with error bars being calculated using standard deviation.

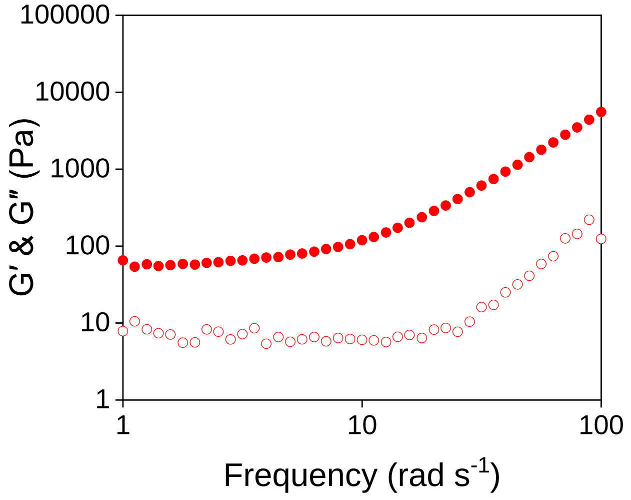

Figure S46. Frequency sweeps of a gel formed from solutions of **PBI-Y** starting at pH 6. Closed circles represent G′ and open circles represent G″. Frequency sweeps were performed at 0.1% strain at 25°C. Data shown is averaged data for triplicate runs of the samples, with the error bars being calculated using standard deviation.

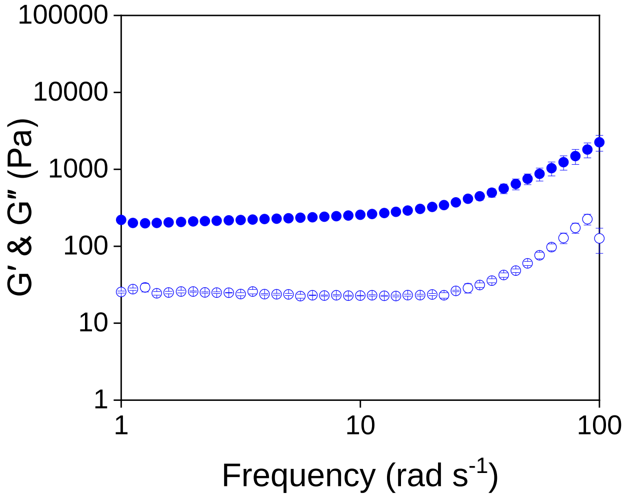

Figure S47. Frequency sweeps of a gel formed from solutions of **PBI-Y** starting at pH 9. Closed circles represent G′ and open circles represent G″. Frequency sweeps were performed at 0.1% strain at 25°C. Data shown is averaged data for triplicate runs of the samples, with the error bars being calculated using standard deviation.

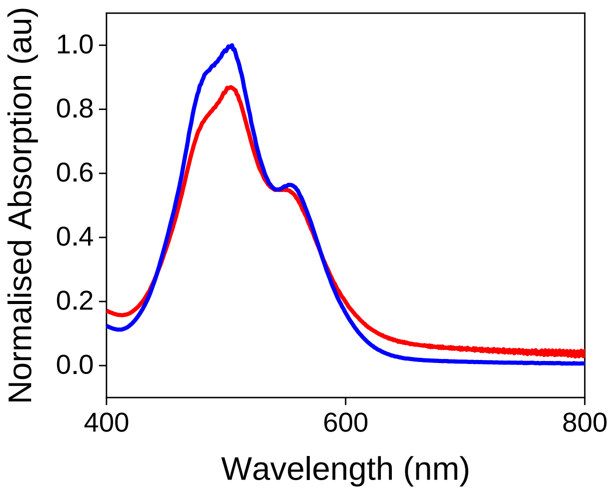

Figure S48. UV-vis absorption spectra of **PBI-L** gels formed from solutions starting at pH 6 (red) and pH 9 (blue).

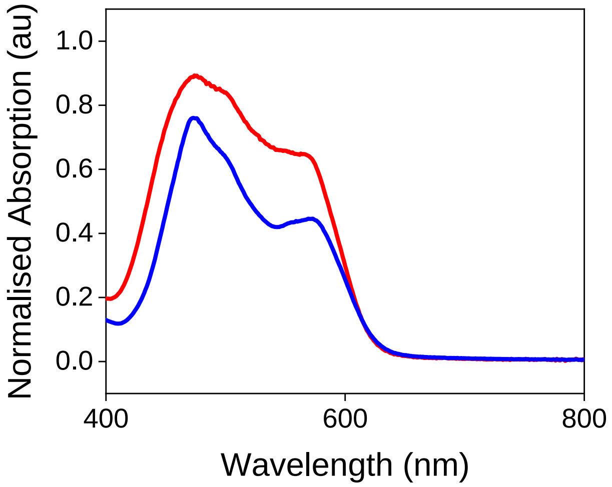

Figure S49. UV-vis absorption spectra of **PBI-V** gels formed from solutions starting at pH 6 (red) and pH 9 (blue).

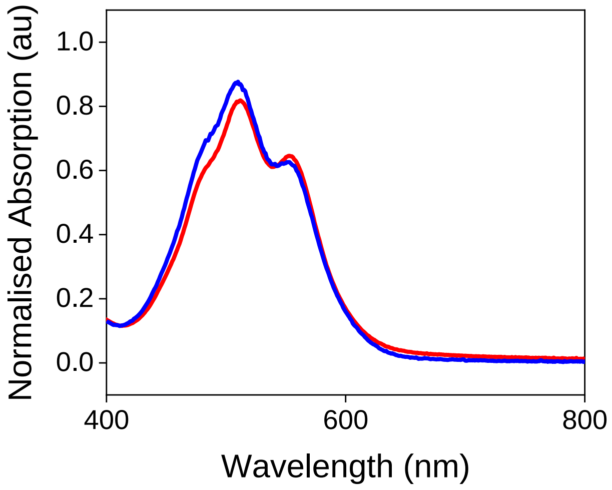

Figure S50. UV-vis absorption spectra of **PBI-Y** gels formed from solutions starting at pH 6 (red) and pH 9 (blue).

3. References

[1] E. R. Draper, J. J. Walsh, T. O. McDonald, M. A. Zwijnenburg, P. J. Cameron, A. J. Cowan, D. J. Adams, *J. Mater. Chem. C* **2014**, *2*, 5570-5575.

[2] O. Arnold, J. C. Bilheux, J. M. Borreguero, A. Buts, S. I. Campbell, L. Chapon, M. Doucet, N. Draper, R. Ferraz Leal, M. A. Gigg, V. E. Lynch, A. Markvardsen, D. J. Mikkelson, R. L. Mikkelson, R. Miller, K. Palmen, P. Parker, G. Passos, T. G. Perring, P. F. Peterson, S. Ren, M. A. Reuter, A. T. Savici, J. W. Taylor, R. J. Taylor, R. Tolchenov, W. Zhou, J. Zikovsky, *Nucl. Instrum.* **2014**, *764*, 156-166.

[3] www.saasview.org

[4] www.ncnr.nist.gov/resources/activation

[5] J. Raeburn, B. Alston, J. Kroeger, T. O. McDonald, J. R. Howse, P. J. Cameron, D. J. Adams, *Mater. Horiz.* **2014**, *1*, 241-246.
